# Altered Gut Microbiome and Fecal Immune Phenotype in Early Preterm Infants With Leaky Gut

**DOI:** 10.3389/fimmu.2022.815046

**Published:** 2022-02-23

**Authors:** Jose M. Lemme-Dumit, Yang Song, Hnin Wai Lwin, Claudia Hernandez-Chavez, Sripriya Sundararajan, Rose M. Viscardi, Jacques Ravel, Marcela F. Pasetti, Bing Ma

**Affiliations:** ^1^ Department of Pediatrics, University of Maryland School of Medicine, Baltimore, MD, United States; ^2^ Center for Vaccine Development and Global Health, University of Maryland School of Medicine, Baltimore, MD, United States; ^3^ Institute for Genome Sciences, University of Maryland School of Medicine, Baltimore, MD, United States; ^4^ Department of Microbiology and Immunology, University of Maryland School of Medicine, Baltimore, MD, United States

**Keywords:** preterm infants, gut microbiome, fecal cytokines, local immune phenotype, intestinal barrier maturation

## Abstract

Intestinal barrier immaturity, or “leaky gut”, is the proximate cause of susceptibility to necrotizing enterocolitis in preterm neonates. Exacerbated intestinal immune responses, gut microbiota dysbiosis, and heightened barrier injury are considered primary triggers of aberrant intestinal maturation in early life. Inordinate host immunity contributes to this process, but the precise elements remain largely uncharacterized, leaving a significant knowledge gap in the biological underpinnings of gut maturation. In this study, we investigated the fecal cytokine profile and gut microbiota in a cohort of 40 early preterm infants <33-weeks-gestation to identify immune markers of intestinal barrier maturation. Three distinct microbiota types were demonstrated to be differentially associated with intestinal permeability (IP), maternal breast milk feeding, and immunological profiles. The *Staphylococcus epidermidis-* and Enterobacteriaceae-predominant microbiota types were associated with an elevated IP, reduced breast milk feeding, and less defined fecal cytokine profile. On the other hand, a lower IP was associated with increased levels of fecal IL-1α/β and a microbiota type that included a wide array of anaerobes with expanded fermentative capacity. Our study demonstrated the critical role of both immunological and microbiological factors in the early development of intestinal barrier that collectively shape the intestinal microenvironment influencing gut homeostasis and postnatal intestinal maturation in early preterm newborns.

## Introduction

A functional intestinal barrier is not just a static physical boundary but a dynamic, interactive tissue structure that combines a cellular barrier with chemical, immunological, and microbiological components (1, 2). The human intestinal epithelium harbors multiple specialized cell types and is supported by a diverse population of underlying immune cells; the tissue arrangement, interaction, and function are controlled by intricate cell contact-mediated signals and distinct cytokines and molecular mediators in the local microenvironment (2, 3).

A community of diverse microorganisms inhabiting the gastrointestinal tract, collectively referred as gut microbiota, contributes to intestinal homeostasis and overall health (4). It is well-accepted that the intestinal microbiota is established concurrently with the developing mucosal immunological barrier after birth (1, 5–8), although it has been argued that the human microbiota may develop even earlier in the prenatal intrauterine environment (9–12). The interactions of microorganisms with immune and non-immune cells that make up the human gut can evoke both an immediate and innate as well as a delayed, and more refined, immune response - a process known as “host-microbial crosstalk” that contains microbial penetration (13). The communications between epithelial cells, immune cells, and gut microbiota orchestrate immune responses to specific antigens and balance tolerance; these processes evolve through the different stages of life to accommodate host developmental needs (2, 7).

Microbial gut dysbiosis (e.g., low microbial diversity), or imbalanced gut microbial community (e.g., overabundance of Proteobacteria), is a common feature of premature intestine compared to term infants (14, 15). Multiple factors appear to be associated with microbial dysbiosis, such as aberrant peristalsis (16), lower gastric acid production (17), altered cell surface glycoconjugates (18), compromised mucin layer and reduced protective molecules (19), and deficient proteolytic enzyme activity (17). In a hyperpermeable infant gut, bacteria and bacterial products normally confined to the intestinal lumen are able to translocate into the inner host compartment, resulting in microbial invasion, mucosal inflammation, epithelial cell damage, necrosis, systemic infection, and ultimately multi-organ failure and death (20–22). The intestinal impairment-associated conditions have a high mortality (6.3%) and morbidity (34.1%) rate; infant survivors may suffer life-long health sequelae such as neurodevelopmental delay, liver failure, or short bowel syndrome (23, 24). In fact, intestinal prematurity is the greatest risk factor for developing necrotizing enterocolitis (NEC) (25, 26), a life-threatening, inflammatory bowel disease affecting approximately 7-10% preterm neonates with mortality as high as 30-50% (27–31). A thorough understanding of microbial and immunological factors that promote intestinal barrier function in early preterm newborns is, therefore, of paramount importance.

The repertoire and magnitude of cytokines and chemokines in preterm newborns remain largely uncharacterized, leaving a significant knowledge gap in understanding early intestinal barrier maturation. The analysis of immunological biomarkers traditionally involves blood or serum testing. Such sampling is invasive and is limited to circulating levels of immune markers, which is valuable for assessment for systemic immunity but not necessarily representative of the localized intestinal mucosa. Intestinal biopsies have been used to determine local microbiome cytokine signatures associated with neurodevelopmental disorder (32). Resected tissue better reflects host-microbe interactions. However, tissue scraping is overly invasive and impractical for studies involving infants. Alternatively, a relevant and non-invasive approach (with easier specimen collection) is fecal profiling of the localized intestinal cytokine microenvironment.

The intestinal immune system is regionally specialized in composition and function, and this distinctiveness has been attributed to anatomical and physiological elements, as well as the inhabiting microbiota (33, 34). Hence, the determination of immune mediators (e.g., cytokines and chemokines) evoked locally as a result of the host interaction with the intestinal microbial community is inherently advantageous to understand both immune quiescence during homeostasis and the inflammatory process that results from an epithelial barrier breach (6).

We previously reported an astonishing 42.5% prevalence of persistently elevated intestinal permeability (IP) in early preterm infants (<33 weeks) (35, 36), with IP being determined by the ratio of urine excretion of ingested sugar (lactulose and rhamnose) probes. Further, this aberrant intestinal maturation was found to be associated with the lack of breast milk feeding, prolonged antibiotics exposure, and perturbation in the development of the intestinal microbiota. In this study, we investigated cytokine and chemokine profiles in fecal extracts obtained from a cohort of 40 early preterm neonates (24-32 weeks of gestation) with both high and low IP at 7-10 days post-birth, to investigate their association with microbiological and neonatal factors implicated in intestinal barrier maturation. Three distinct gut microbiota types were revealed, which were differentially associated with IP and nutritional factors as well as distinct immunological profiles. Most outstandingly, infants with lower IP had increased levels of IL-1α/β and a type of gut microbiota with a more diversified array of anaerobic and facultative bacteria. Our study demonstrated distinct immunological and microbiological features during early stages of development of the intestinal barrier that collectively influence gut homeostasis and postnatal intestinal maturation in early preterm newborns.

## Results

### Intestinal Permeability in Preterm Newborns

Through monitoring IP in previous studies, we and others have demonstrated that rapid intestinal barrier maturation occurs in most preterm infants during the first 7-10 days after birth, despite highly permeable gut at birth in most newborns (35–37). IP was determined as the ratio of two enterally administered sugar probes Lactulose (La) and Rhamnose (Rh), which are markers of intestinal paracellular and transcellular pathways, respectively (38, 39). High IP was defined as La/Rh ratio >0.05, as previously described and validated (35). Persistently elevated IP during the 7-10 days after birth indicated a delayed physiological maturity of the intestinal tract barrier in early preterm infants. In this study, we employed a cohort of 40 early preterm newborns 24 weeks (24^0/7^)-32 weeks and 6 days (32^6/7^) of gestation recruited at the Neonatal Intensive Care Unit (NICU) of University of Maryland Medical Center in Baltimore, Maryland. Twenty of these infants had high IP while the remaining had low IP 7-10 days after birth. Metagenomic sequencing was performed on fecal samples obtained from these 40 infants between 7 to 10 postnatal days. The IP values for the cohort ranged from 0.001 to 0.11 with an average of 0.09 ± 0.09 (mean ± SD). Cohort subjects were 30.2 ± 2.43 weeks gestational age (GA) at birth. Their mean birth weight (BW) was 1,414 g ( ± 414.6 g); 21 (52.5%) neonates were classified as very low BW (VLBW, <1,500 g) and 8 (20.0%) were classified as extremely low BW (ELBW, <1,000 g). Sixteen of the infants (40.0%) were born with spontaneous vaginal delivery, the other 24 infants were delivered by cesarean section. The demographic, obstetric, and neonatal characteristics of the subjects are summarized in **Supplemental Table 1**.

### Microbiome Type Correlates With Intestinal Permeability and Maternal Breast Milk Feed

**Figure 1:**
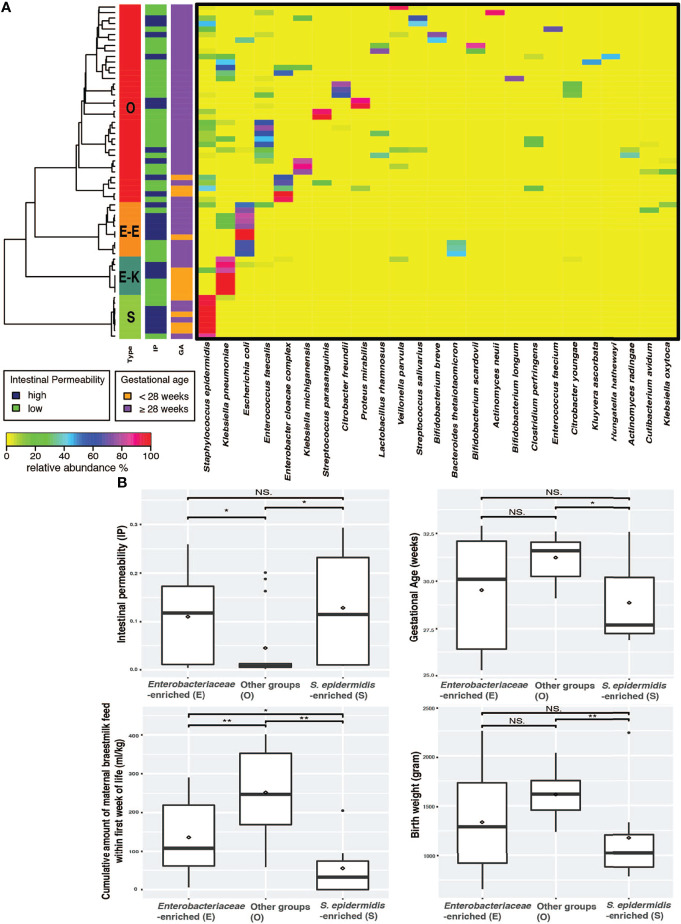
Preterm infants exhibited three distinct microbiome types. **(A)** Heatmap of the 25 most abundant intestinal bacterial taxa and their relative abundance in samples collected from 38 preterm infants enrolled in the study. The taxonomic composition of the microbiomes used the data set of whole community metagenomic sequencing and the profile was generated by MetaPhlAn version 2 (40). Statistical significance was calculated using Wilcoxon rank sum test using *ggsignif* R package (41). Ward linkage clustering was used to cluster samples based on their Jensen-Shannon distance calculated in vegan package in R (42). The number of clusters was validated using gap statistics implemented in the *cluster* package in R (43) by calculating the goodness of clustering measure. **(B)** Boxplots of IP, GA, MOM, and BW depicting distribution of microbiota types in fecal samples of preterm newborns. Significance value was calculated using Wilcoxon rank sum test. *p < 0.05, **p < 0.01. Plotted are interquartile ranges (IQRs, boxes), medians (line in box), and mean (red diamond). IP, intestinal permeability; GA, gestational age; NS., not significant.

The gut microbial community was determined by conducting whole community metagenomic sequencing of 61 fecal specimens collected from the 38 infants; 18 of them had more than one fecal sample collected during the 7-10 days after birth. A total of 1,721.8 million metagenomic sequence reads (average of ~28.2 million sequence reads per sample) were obtained after quality assessment (**Supplemental Table 2**). Taxonomic profiles were determined for all samples (**Supplemental Table 3**) and clustered into three distinct groups according to similarities in community composition and structure (**Figure 1A**). Two of these groups have a single species that is dominantly abundant (>95%): the *Staphylococcus epidermidis*-predominated community type (the “S” type), and Enterobacteriaceae (the “E” type) predominated by either *Klebsiella pneumoniae* or *Escherichia coli*. On the other hand, the “O” type of gut microbiota encompasses a wide array of anaerobic and facultative microorganisms and was not predominated by *S. epidermidis*, *K. pneumoniae* or *E. coli*. The O type demonstrated a significantly higher community bacterial diversity (p-value < 0.05) than the S type, which has the lowest level of bacterial diversity (**Figure 1A** and **Supplemental Figure 1**). The predominantly abundant taxa of the E and S types were shown to be Enterobacteriaceae (i.e., *K. pneumoniae* or *E. coli*) and *S. epidermidis*, respectively. The top abundant taxonomic groups of O type include *Bifidobacterium*, *Lactobacillales*, *Veillonellales*, *Clostridiales*. These data support our previous findings that intestinal barrier maturation correlates with the establishment and colonization of a diverse array of obligate and facultative anaerobes, particularly in the groups of *Actinobacteria*, *Lactobacillales*, *Veillonellales*, and *Clostridiales*, which are considered the ‘successor’ bacteria to the first colonizers *S. epidermidis* and Enterobacteria, during the establishment of commensal communities after birth (36, 44, 45).

We next examined the correlation of the three microbiota types (E, S and O) with IP and the known IP-associated factors that include GA, BW, mother’s own breast milk (MOM) feed, and antibiotics (abx) duration (35, 36). Delivery mode was also associated with IP; infants delivered through cesarean section had significantly higher IP than those delivered through spontaneous vaginal birth (p value = 0.01, **Supplemental Figure 5**). The O microbiota type was associated with newborns that had low IP, later GA (≥ 28 weeks), higher BW (≥ 1,500g), and more cumulative amount of MOM (≥ 150-180 ml/kg of cumulative intake) by 7-10 postnatal days (**Figure 1B**). The S microbiota type, on the other hand, correlated with high IP, early GA (< 28 weeks), lower BW (< 1,500g), and less MOM (<150 ml/kg of cumulative intake). Preterm infants with E microbiota type had significantly higher IP than those with O type but not those with S type and high MOM intake. Further, GA and BW were similar in E and S type microbiota (**Figure 1B**).

### Fecal IL-1α and IL-7 Correlated With Increased Microbial Biodiversity

**Figure 2:**
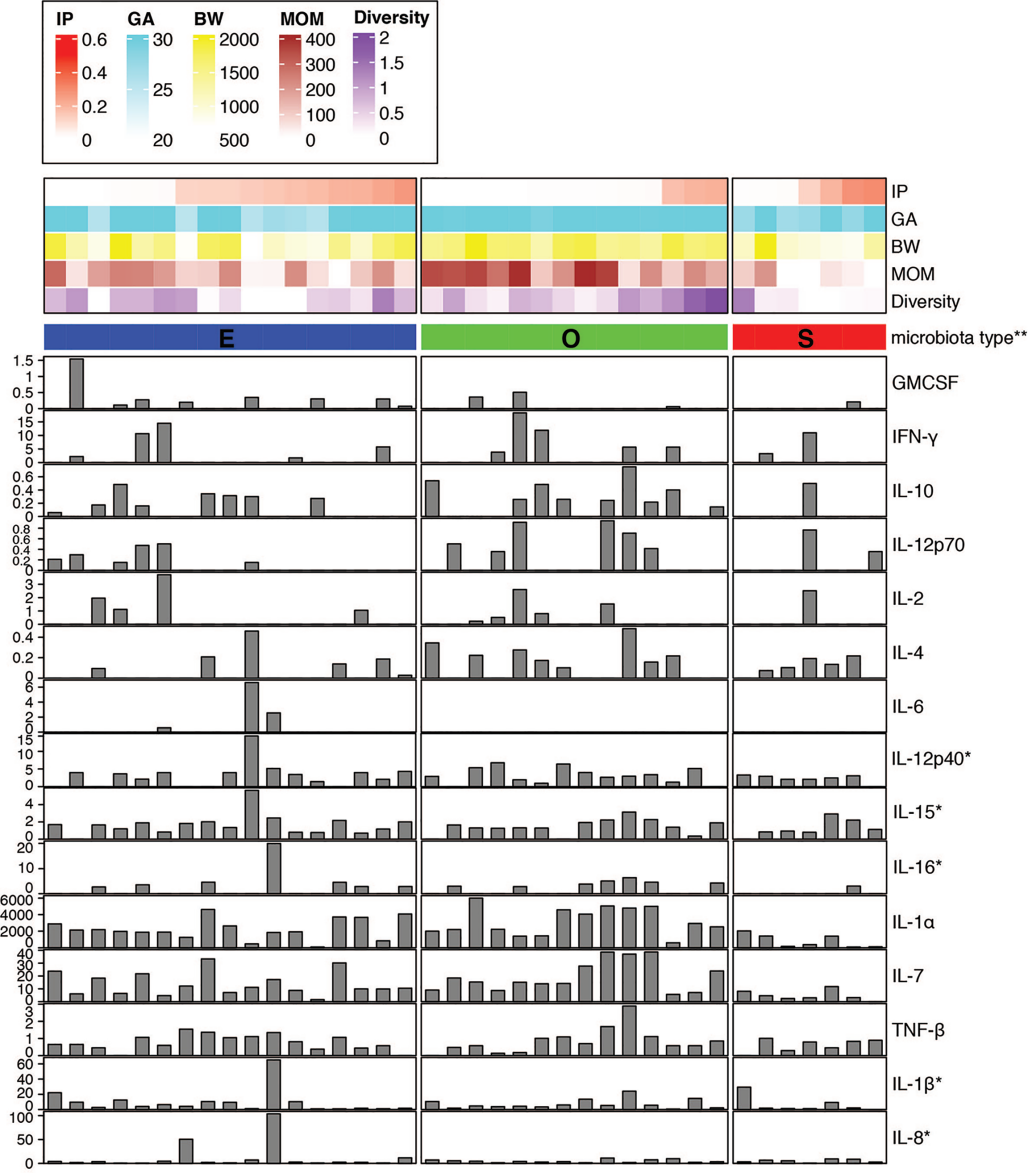
Fecal cytokine profile associated with microbial diversity. Color map of microbial communities correlating with neonatal factors and barplot map of the 15 cytokines detected. Within-sample diversity was estimated using Shannon diversity index using Phyloseq R package (46). Plot was generated using R package ‘complexheatmap’ (47). *Value was scaled using square root. **Microbiota type was assigned according to the clustering pattern as shown in **Figure 1**. IP, intestinal permeability; GA, gestational age; BW, birth weight; MOM, mother’s own breast milk cumulative volume use during the first week prior to IP measurement.

We sought to investigate the profile of cytokines and chemokines in the fecal samples from preterm infants as markers of gut inflammation and mucosal adaptation to intestinal microbiota (**Figure 2** and **Supplemental Table 5**). Given the lack of information on local mucosal immune mediators in preterm infants, a broad panel of 16 cytokines and 14 chemokines produced and secreted by intestinal epithelial cells and immune cells was selected. The immune biomarkers were analyzed considering neonatal and nutritional factors including IP, GA and BW, MOM feed, and gut microbiota type. Out of a total of 30 cytokines and chemokines measured, 15 of them were detected, whereas the others were undetectable or below the limit of detection for the immunoassay. Strikingly, infants that exhibited higher IP and medium-to-low MOM intake presented reduced or undetectable levels for most of the cytokines independent of the gut microbiota type (**Figure 2**). This finding emphasizes the differences between the immature and mature intestine, and the distinctiveness of the preterm population as compared to term infants and adults.

Stool of infants with O type gut microbiota contained significantly higher levels of IL-1α and IL-7 as compared to S type samples (**Figure 2** and **Supplemental Figures 2A, B**). In a correlation analysis with individual species abundance using linear regression, IL-1α and IL-7 were shown to be both positively correlated with the relative abundance of *Enterococcus faecalis* and negatively correlated with *S. epidermidis* (**Supplemental Figure 3**).

Further, significantly increased levels of IL-12p70 were detected in O type microbiota as compared to E type (**Supplemental Figure 2C**). The presence of other cytokines including IL-12p40, IL-15, and IL-16 were also higher in O type specimens in comparison to S type group (**Figure 2**); whereas IL-6 was detected only in the stool of E type microbiota as compared to S and O type groups (**Figure 2** and **Supplemental Figure 2D**).

### IL-1β and IL-1α Association With Low Intestinal Permeability

**Figure 3:**
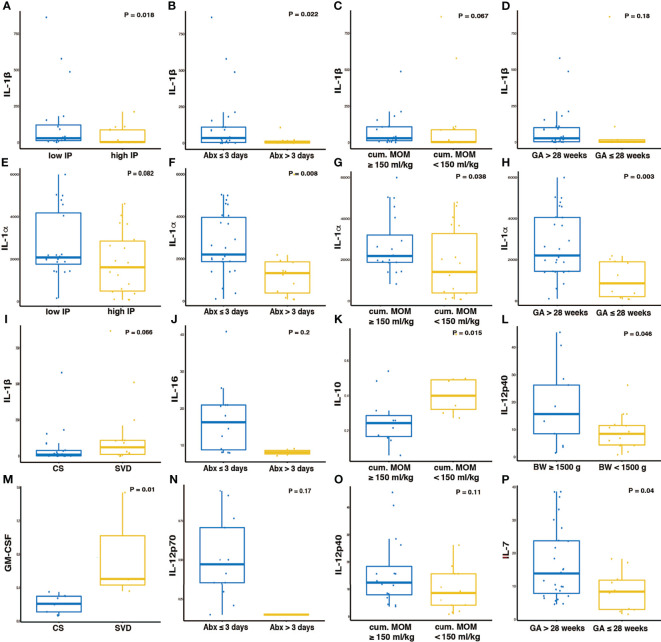
IL-1α and IL-1β linked to lower IP and improved neonatal factors. Boxplots of the cytokine levels: **(A-D, I)** IL-1β, **(E–H)** IL-1α, **(J)** IL-16, **(K)** IL-10, **(L, O)** IL-12p40, **(M)** GM-CSF, **(N)** IL-12p70, **(P)** IL-7 between different categories. Plotted are interquartile ranges (IQRs, boxes), medians (line in box), and mean (red diamond). Significance value was calculated using Wilcoxon rank sum test using *ggsignif* R package (41). Asterisk denotes the level of significance. Threshold for early or late gestational age is 28 weeks, for low or high birth weight is 1,500 g, for longer or shorter abx exposure is 3 days during the first week after birth prior to IP measurement, for maternal breast milk feeding is 150-180 ml/kg of cumulative intake of MOM by 7-10 days of age, according to clinical convention and previously validation (35, 36). IP, intestinal permeability; GA, gestational age; BW, birth weight; MOM, mother’s own breast milk (MOM) cumulative volume use during the first week prior to IP measurement; SVD, spontaneous vaginal delivery; CS, cesarean section.

Comparative analysis of fecal cytokines in samples with high (La/Rh ≥0.05) or low IP (La/Rh <0.05) revealed a significant correlation between IL-1β and low IP (p-value < 0.01) (**Figure 3A**). IL-1β was also associated with less use of abx (≤ 3 days, p-value < 0.05) (**Figure 3B**). IL-1β levels showed a trend for correlation with MOM feed (p-value=0.067) (**Figure 3C**), while it did not associate with GA (**Figure 3D**). IL-1α levels showed a statistical trend for association with low IP (p-value=0.08) (**Figure 3E**) and was significantly increased in infants with less abx (p-value < 0.01) (**Figure 3F**), more MOM feed (p-value <0.05) (**Figure 3G**), and later GA (p-value < 0.01) (**Figure 3H**). These data suggest an important role for IL-1β and IL-1α in the multifactorial processes involved in enhanced intestinal barrier and immune maturity. IL-1α was more closely linked with age and birth weight and may represent an immunological biomarker for age-appropriate intestinal maturation. On the other hand, IL-1β levels are most significantly correlated with IP that is irrespective of age, which suggests a potential influence in microbiota development. Still, postnatal intestinal maturation is a complex process that involves multiple factors that shape the infant gut environment, including the nervous system and mucosal lymphoid tissue development, and host-microbe interactions in addition to early microbial colonization (30, 48–50).

When compared with the abundance of individual species, IL-1β was found to be positively correlated with *Bacteroides* and *Clostridium perfringens* (**Supplemental Figure 3**). IL-16 and IL-12p70 levels showed a trend for correlation with less abx intake (p-value=0.2) (**Figures 3J, N**). Strikingly, IL-10 was significantly increased in infants with less MOM feed (**Figure 3K**) and negatively correlated with the relative abundance of *Peptostreptococcus anaerobius* and *Veillonella parvula* (**Supplemental Figure 3**). Further, IL-12p40 was significantly associated with greater BW (**Figure 3L**) and found to be negatively correlated with *S. epidermidis* relative abundance (**Supplemental Figure 3**), which suggests that IL-12p40 may be involved in the reduction of *S. epidermidis* during microbial succession and the establishment of infant immunity, along with IL-1α and IL-7. Increased levels of IL-1β showed a statistical trend for association with spontaneous vaginal delivery (p-value=0.066) (**Figure 3I**), while granulocyte-macrophage colony-stimulating factor (GM-CSF) were significantly associated with this delivery mode (p-value=0.01) (**Figure 3M**). There was a trend of reduced IL-12p40 levels in infants with lower MOM feed (**Figure 3O**). IL-7 was significantly associated with later GA (p-value < 0.05) (**Figure 3P**).

### High Inter-individual Variation of Cytokine Profile in S. epidermidis- and Enterobacteriaceae-Dominated Microbial Communities

**Figure 4:**
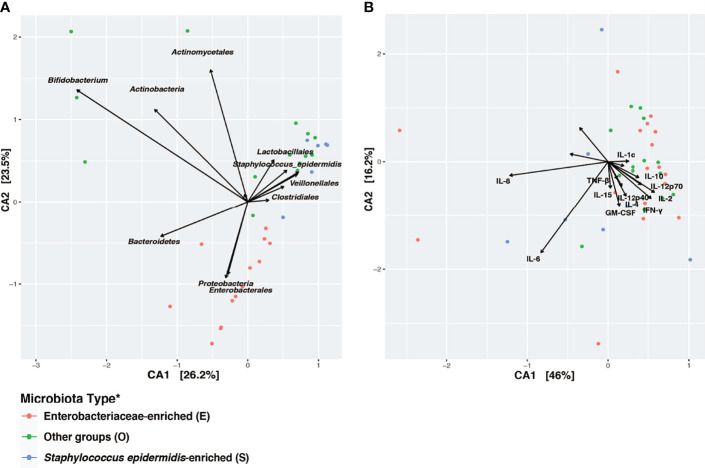
Disparity in clustering patterns of taxonomic and immunological profiling among different microbiota types. Canonical Correspondence Analysis (CCA) of **(A)** microbial taxonomic groups; **(B)** cytokine profiles. CCA was based on Bray-Curtis distance. CA1 and CA2 selected as the major components based on the eigenvalue. A scaled eigenvalues was shown on the plot to represent the direction from the origin where a group has a larger than average value for the particular profile (42, 51). *Microbiota type was assigned according to the clustering pattern demonstrated in **Figure 1**.

**Figure 5:**
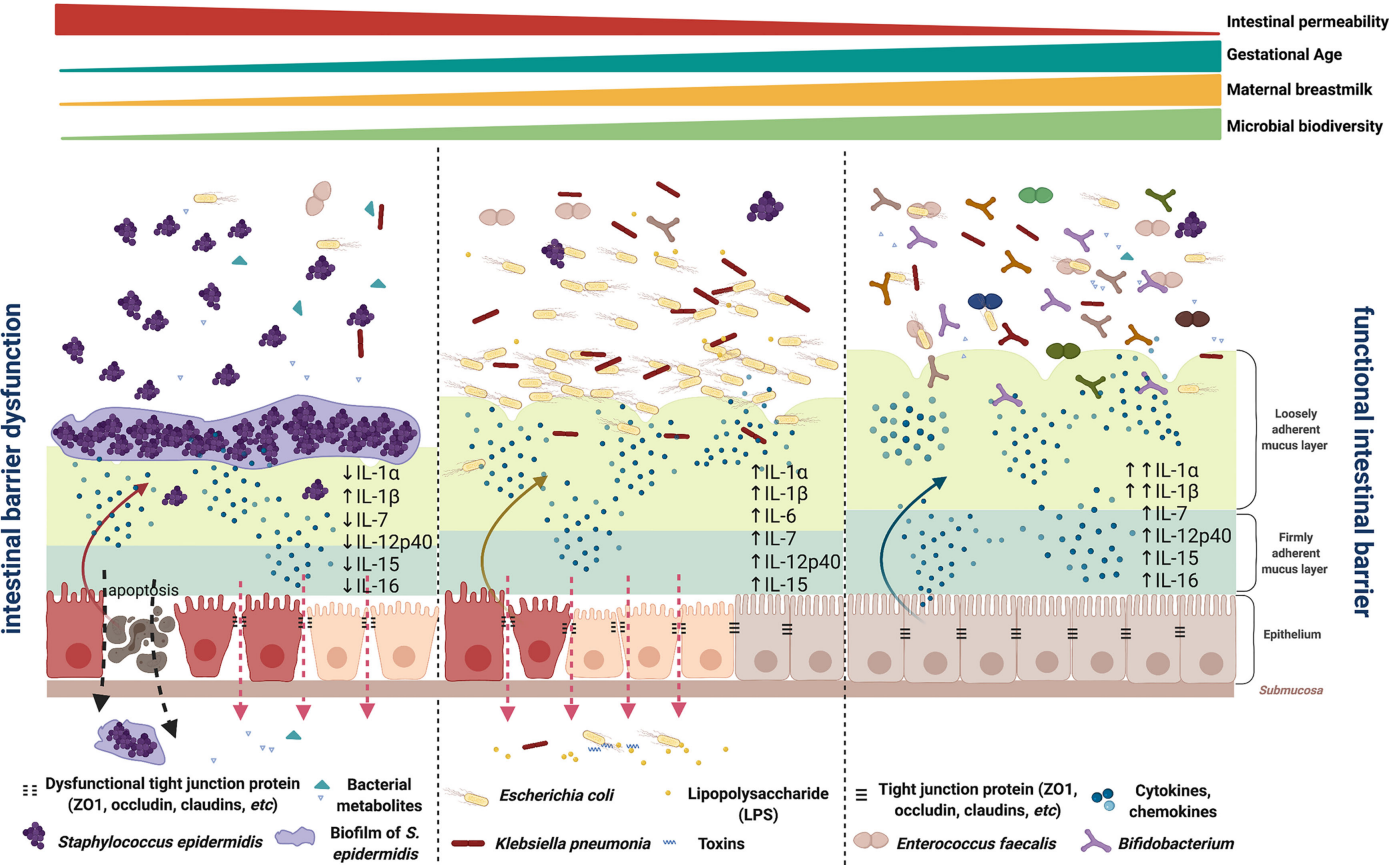
Illustration of the intestinal barrier maturation in early preterm neonates with different microbiota types and cytokine profiles. An immature, compromised gut barrier may render the mucosa susceptible to invasion by opportunistic pathogens in the gut lumen. IP was linked with a microbial community dominated by a single species *S. epidermidis*, *K. pneumoniae*, or *E. coli*, with less defined cytokine profiles among individuals. A functional intestinal barrier was associated with neonates with later GA; higher BW; greater microbial community biodiversity that encompasses a wide array of anaerobic and facultative microorganisms not dominated by *S. epidermidis*, *K. pneumoniae*, or *E. coli*; and a trend of increased levels of IL-1α/β, IL-7, IL-12p40, IL-15, and IL-16. Schematic representation illustrates the distal intestine (not drawn to scale). Created with BioRender.com.

Canonical Correspondence Analysis (CCA) on the taxonomic profiles further supported the presence of three distinct microbiota types, as described above (**Figure 4A**). Based on a scaled eigenvalue, the top taxonomic groups that contributed to the separation of the microbiota type in ordination analyses were identified (**Supplemental Table 4**). Both *S. epidermidis-* and Enterobacteriaceae-dominated microbial community shared a greater within-cluster similarity, or a “tight” clustering based on taxonomic (**Figure 4A**) and functional pathway profiles as determined by metagenomes (**Supplemental Figure 4**). CCA was also applied to visualize the patterns of gut microbial and fecal cytokine profiles in the preterm infants (**Figure 4B**). Both the S and the E microbiota types, particularly the *S. epidermidis*-dominated type, had a highly diversified immunological profile with much greater inter-individual variation that is, a “loose” cluster based on immune markers (**Figure 4B**). Conversely, the O type microbiota had a more diverse clustering of taxonomic groups and more diversified functional profiles and greater within-cluster similarity based on cytokine and chemokine profiles. These results indicate that host immunity to S or E microbiota types varies markedly among individuals. This inter-individual variation in immune profiles is indicative of an unstable and less defined immunological environment, which may relate to a lack of establishment of age-appropriate gut microbiota (**Figure 5**). These data suggest that as the intestinal microbiota becomes established with an age-appropriate bacterial colonization, the mucosal immunity in the preterm infants tend to be more stable and defined. Our findings highlight the importance of defining both immune profiles and microbiome to holistically understand postnatal intestinal maturation.

## Discussion

The pathophysiology of aberrant postnatal intestinal maturation is a multifactorial process that includes intestinal mucosa barrier immaturity, imbalance in microvascular tone, aberrant microbial colonization, and altered immune responses (30, 48, 49). We and others have previously shown that neonatal and clinical factors including GA or postmenstrual age, abx exposure, and exclusive breast milk feeding were associated with intestinal barrier function in early preterm infants (35, 52). Further, a delayed increase in intestinal bacterial biodiversity due to a lack of breast milk-derived or -promoted growth of *Bifidobacterium*, was shown to contribute to delayed growth and delayed intestinal maturation (35, 36, 53, 54). In this study, we sought to delineate the complex mechanisms governing intestinal barrier maturation by characterizing fecal cytokines and chemokines and interrogating correlates with IP-associated neonatal and microbiological factors. Integrative insights into the interplay between gut microbiota and the infant immune system are crucial to understanding the underpinning of intestinal immaturity and to inform the development of novel therapeutics to prevent leaky gut in premature newborns.

Cytokines and growth factors are important mediators of intestinal epithelial cell development, differentiation, expansion, and immune activation (55). They are expressed endogenously by the midgestational intestine at higher levels as compared to the adult counterpart (56). After birth, the newborn and infant intestine continue to be influenced by cytokines and growth factors from colostrum and breast milk. Cytokine profiles are typically determined as circulating levels in serum samples. However, such measurements fail to reflect local cell-signaling and immune mediators. The profiling of cytokines in fecal extracts offers a snapshot of the mucosal intestinal environmental. While the approach may not fully recapitulate the host-microbe interactions that occur at the epithelial surface, it provides a reasonable and practical alternative to exceedingly invasive tissue sampling (e.g., biopsy, mucus biofilm sampling or tissue scrapping) which is neither practical nor feasible in routine pediatric studies. The differential abundance of specific gut bacteria has been associated with distinct cytokine responses, and this effect was exerted directly on the intrinsic cytokine production capacity of the immune cells rather than by influencing the number of cells in circulation (57). Profiling of stool cytokines represents a promising, non-invasive method to investigate the localized intestinal cytokine microenvironment, and when combined with microbiota data, it can be a powerful and practical tool to study neonatal intestinal disorders.

Numerous studies have documented an intestinal microbial succession that begins soon after birth (36, 58–60). The initial colonization right after birth includes facultative anaerobes, e.g., *S. epidermis* and Enterobacteriaceae, which are considered “first colonizers” of the infant gut. It is then rapidly followed by the accumulation of obligate anaerobes, including *Bifidobacterium*, *Bacteroides*, and *Clostridiales* with expanded fermentative metabolism capacity. Progressive succession of the early-life microbiota was shown to influence growth and maturation of the endocrine, mucosal immune, and central nervous systems (61). An imbalanced microbiome has been associated with increased risk of neonatal pathologies, including NEC and late-onset sepsis (LOS) (62). Infants with NEC are more likely to develop LOS mainly due to the translocation of intestinal bacteria across the epithelial barrier and the ensuing inflammation (63, 64). *S. epidermis* was shown to diminish rapidly after birth and is usually replenished by obligate anaerobes (36, 58–60). Persistent predominance of *S. epidermidis* and delayed or aberrant microbial succession was associated with growth impairment and predisposition to adverse health conditions (65, 66). *S. epidermidis* is actually the leading agent causing neonatal LOS and other inflammation-related neonatal conditions in preterm neonates including bronchopulmonary dysplasia, and the severity of these conditions is often underestimated (67–71). Though the mechanisms remain unclear, prematurity increased susceptibility of newborn pigs to *S. epidermidis*-associated sepsis, and mortality was related to an immature immune system (72). In the present study, the high IP-associated *S. epidermidis-*predominant community had the greatest inter-individual variation in cytokine responses among all three microbiota types, suggesting an unstable, inordinate mucosal immunity in the immature intestine microenvironment. Overall, our data demonstrate the three stages of microbial succession in early preterm infants, and most importantly, their associations with intestinal immunity during early intestinal maturation (**Figure 5**). The characterization of interactions between early microbial colonization and host immunity as well as their synergistic responses to IP-associated factors such as nutrition, age, and abx intake is pivotal to understand the mechanisms involved in postnatal epithelial barrier maturation.

The ability to derive energy from diet is important for microbial succession and shifting away from Bacilli or Enterobacteriaceae-predominant communities (65, 66, 73). In fact, the diversity of the microbiota remains low in early infancy and is dominated by species involved in human milk oligosaccharide (HMO) metabolism in breastfed infants. In the current study, we demonstrate that the O type microbiota, indicative of optimal microbial succession with a wide array of bacteria with increased fermentative capacity (i.e., *Bifidobacterium*, *Lactobacillus*, *Bacteroides*), is associated with improved intestinal barrier functions and distinct cytokine markers, primarily IL-1α/β.

Human milk is a rich source of nutrients and bioactive molecules including immunoglobulins, cytokines, and immune cells, which support tissue development and protect infants against infectious agents (74). We recently reported that human breast milk enhanced intestinal barrier function and ameliorated pro-inflammatory cytokines production in a human pediatric enteroid model (75). This beneficial effect appears to be mediated by HMO-utilizing *Bifidobacterium* species, whose abundance, we found, correlates with improved intestinal barrier integrity and which is genetically equipped to digest these nutrients. Further characterization of the interaction between HMOs’ metabolism, intestinal microbiota, and immunological response will be important to obtain a detailed understanding of postnatal intestinal barrier maturation.

Clearly, the cytokine profile of a mature gut cannot be compared to the profile of the highly immature intestine in the early developmental stage. Increased levels of IL-1α/β in preterm infants were shown to be positively correlated with neonatal factors associated with improved intestinal barrier functions. While the IL-1 superfamily of cytokines has been linked with intestinal barrier dysfunction and gut inflammation in adults, these cytokines have pleiotropic functions, including support of gut homeostasis through various mechanisms (76). For instance, macrophage-derived IL-1β induces IL-2 secretion by ILC3 cells, and IL-2 is essential for maintaining Treg cells, immunological homeostasis, and oral tolerance to dietary antigens in the small intestine as shown in an *in vivo* mouse model (65). In addition, IL-1β, among other cytokines, stimulates the expression of polymeric immunoglobulin receptor and IgA transcytosis across a primary murine epithelial cell monolayer (77). Further, our results suggest that IL-1α and IL-1β relate to intestinal barrier function in a dichotomous way; IL-1β was most closely associated with IP, while IL-1α appeared to be correlated primarily with age-appropriate microbiota development. A previous study suggested that the production of IL-1β was regulated largely at a genetic level and less influenced by microorganisms (78). Together, our results suggest a beneficial role for IL-1α/β in maintaining gut homeostasis during early development of immature gut. Follow-up studies with in-depth mechanistic interrogation are warranted.

Other cytokines detected in fecal samples and positively correlated with low IP and with other neonatal factors included IL-7, IL-16, and IL-12p40. IL-7 is recognized as an essential factor for lymphopoiesis (79). Intestinal epithelial cells supply local IL-7, as shown by mRNA expression and immunohistochemistry analyses of human intestinal biopsies, and exogenous addition of recombinant IL-7 to isolated lamina propria lymphocytes suggested that locally sourced IL-7 regulated intestinal lamina propria lymphocyte expansion (80). A mechanistic interrogation of IL-7 functions in transgenic mice showed that a dysregulation of colonic epithelial cell-derived IL-7 resulted in chronic inflammation associated with decreased frequencies of γδ T cells and CD8αα^+^ cells in the inflamed area (81). In an infection model of *Citrobacter rodentium*, intestinal mucosa-derived IL-7 protected the host by controlling bacterial burden and intestinal damage (82). IL-7 stimulates extrathymic γδ T cell precursors and aggregation in cryptopatches in the intestinal mucosa (83). Intraepithelial lymphocytes (IELs), an heterogenous lymphocyte population comprised of γδ T cells among other subsets with anti-microbial and homeostatic functions, reside within the epithelial cell layer of the human gut and strengthen the mucosal barrier (84). IELs appear early in life and gradually accumulate with age after exposure to exogenous antigens (84). In our study, the presence of IL-7 in infants of late GA and less permeable intestine is consistent with expansion of gut γδ IELs during the perinatal period, which in turn promote epithelial cell proliferation and maturation (85). Once established in tissue, γδ IEL relies on the production of IL-15 by the intestinal epithelial cells (86). Our findings support IL-15 production in a microbiota-dependent manner which promotes IEL localization within the intestinal mucosa and function. In the absence of these barrier-enhancing factors, the infiltration of microbes could lead to local necrosis (i.e., NEC and LOS). Future mechanistic studies interrogating the role of IL-7 in the developing infant gut are warranted.

IL-16 is secreted by a variety of cells including lymphocytes and epithelial cells; it functions as chemoattractant of CD4^+^ cells and triggers the production of pro-inflammatory cytokines by human monocytes (87, 88). Elevated levels of IL-16 have been reported in patients with inflammatory bowel disease and neonatal sepsis (89, 90). In the present study, a trend of increased levels of IL-16 was observed in infants with less abx exposure, reflecting local inflammation. We have also seen increased levels of IL-12p40 positively correlated with infant BW. IL-12p40 has a regulatory function; it competes with IL-12p70 for binding to the IL-12 receptor and is a chemoattractant of macrophages and dendritic cells (91). In an *in vivo* mouse model, IL-12p40 has been shown to limit progression of chronic inflammation by suppressing mucosal Th1 and Th17 responses *via* hypoxia-inducible factor-1α (92).

Unpredictably, IL-10 was inversely correlated with maternal milk feeding. IL-10 is a potent anti-inflammatory and immunosuppressive cytokine; an abundance of IL-10 may limit or downregulate host immune response to microbes (93). The presence/roles of IL-12p40 and IL-10 might be intertwined as compensatory. IL-12p40 production by LPS-stimulated human peripheral blood cells was found to increase following IL-10 blockade (94). In human neonatal cells, TCR/IL-12 stimulation downregulated the IL-10 pathway (95). Together, the local cytokines identified reflect intestinal barrier maturation as well as mucosal immune fitness and aptitude for immunosurveillance.

A study of cytokines in umbilical cord of healthy term newborns found no differences in the levels of inflammatory mediators when comparing normal spontaneous delivery vs. elective cesarean section, except for TGF-β1 (96). Others had reported increased circulating levels of IL-1β in neonates delivered vaginally as compared to those delivered by C-section (97). Similarly, we found that spontaneous vaginal delivery was associated with increased fecal IL-1β and GM-CSF of preterm infants, which suggests that gut maturation and mucosal immune cell seeding may require additional molecular drivers.

Given that cytokines were measured in the feces of breastfeeding infants, the presence of cytokines of maternal origin can be a confounder in the interpretation of infant responses (98). Future studies with larger sample size and longitudinal sampling of both infant and maternal specimens to capture the dynamics and evolution of microbes and immune features are warranted. Additionally, the immune modulatory effect of the microbiota may be exerted through the release of intermediary common mediators such as bacterial products or bioactive bacterial metabolites. The impact of metabolic activities of the microbial community in host-microbe interactions and immunological development remains to be elucidated. Likewise, the observed association between postnatal intestinal maturation and mode of delivery requires further validation. Comprehensive understanding of the role of gut cytokines, chemokines, and other molecular immune mediators in promoting intestinal homeostasis and in mitigating perturbation at early stages of intestinal development, will inform future targeted modulation to improve overall infant health.

## Conclusion

Our study revealed a pattern of fecal cytokines associated with specific changes in gut microbiota and intestinal barrier maturation. Our findings revealed, for the first time, a potential beneficial role of IL-1α/β in age-appropriate microbiota development and low intestinal permeability in early preterm infants. A delayed microbiota maturation was associated with persistently elevated IP, less breast milk feeding, early GA and low BW, as well as inordinate cytokine profiles. These results support the promising, practical, and non-invasive analysis of cytokine and chemokine profiles in fecal samples to define immune phenotypes associated with gut maturation. A detailed definition of the factors affecting the early development of intestinal barrier functions and precise molecular mechanisms underlying gut homeostasis is necessary to prevent life threatening hyperpermeable gut-associated conditions, including NEC.

## Methods and Materials

### Participants Description and Feeding Protocol

This study was approved and carried out in accordance with protocols approved by the institutional review boards of the University of Maryland School of Medicine and Mercy Medical Center. Written informed parental consent was obtained for all infants in accordance with approved protocol UMB HP-00049647. Forty preterm infants 24^0/7^-32^6/7^ weeks GA were enrolled within 4 days after birth, and they represent combined cohorts enrolled during October 2018-June 2021. Prior to study procedures, a complete physical exam including infant’s vital signs, weight, height, and head circumference was performed. Demographic, obstetric, and clinical data, as well as data on medication exposures, feeding practices, and adverse events were collected from the medical record. Threshold for early or late gestational age is 28 weeks, for low or high birth weight is 1,500 g, for longer or shorter antibiotics exposure is 3 days during the first week of life prior to IP measurement, as described previously (35, 36).

A standard feeding protocol was used for all preterm participants. Enteral feeds by the orograstric or nasogastric route were initiated between the first and fourth day of life depending on clinical stability. After initial feeds of 10 ml/kg expressed breast milk or 20 kcal/oz preterm formula or donor breast milk daily for 3-5 days, feedings were advanced by 20 ml/kg/d until 100 ml/kg/d was reached. The total volume of breast milk feeds was calculated as sum of the daily amount of milk intake per kilogram of the administered expressed mother’s breast milk from initial feed day till postnatal day 7-10 when the IP was measured. Feedings were held or discontinued if there were signs of feeding intolerance such as abdominal distension, gastric residuals, or hematochezia, or for clinical deterioration. Maternal breast milk feeding threshold is 150-180 ml/kg of cumulative intake of MOM by 7-10 days of age, as described previously (35, 36).

### *In Vivo* Intestinal Permeability (IP) Measurement

Among the 40 subjects used in this study, IP for 26 of them was measured in a previous study (35, 36); 14 eligible preterm infants received 1 ml/kg of the non-metabolized sugar probes on postnatal day 7-10, which included lactulose (La, Cumberland Pharmaceuticals, Nashville, TN), which is the marker of intestinal paracellular transport and rhamnose (Rh, Saccharides, Inc., Calgary, Alberta, Canada) the marker of intestinal transcellular transport. One milliliter of 8.6 g La and 140 mg Rh per 100 mL solution was administered enterally by nipple or by gavage *via* a clinically indicated orogastric tube (99). La/Rh was measured by high-pressure liquid chromatography (HPLC) in urine collected over a 4h period following administration of the sugar probes as previously described (35). High or low IP was defined by a La/Rh >0.05 or ≤0.05 respectively, as validated and applied previously (35). Urine samples were stored at -80°C until processed.

### Fecal Sample Collection and Processing

Dry fecal samples (~1g) were collected during the same time period (postnatal day 7-10) as the dual sugar administration. Fecal specimens were placed immediately in sterile tube containers with attached screw cap and collection spoon (Globe Scientific Inc. Mahwah, NJ) and stored in -80°C freezer. Another aliquot of fecal samples were stored immediately in DNA/RNA shields (Zymo Research, Irvine, CA), which stabilizes and protects the integrity of nucleic acids to minimize the need to immediately process or freeze specimen for DNA extraction and metagenomic sequencing. Specimens were stored at -80°C until processed.

### Cytokine and Chemokine Measurements

A 100 mg aliquot of each frozen dry fecal specimen was resuspended in 1 ml of extraction buffer [phosphate buffered saline (PBS, pH 7.4) containing 0.01% soybean trypsin inhibitor, 0.1% ethylenediaminetetraacetic acid, and 0.05% Tween 20, all from Sigma, St. Louis, MO] and ~1.5 g of 2.3 mm zirconium beads (Biospec, Bartlesville, OK) were incorporated. Fecal samples were subjected to 1-min beating cycle in a mini-beadbeater-8 tissue homogenizer (Biospec, Bartlesville, OK) and centrifuged at 14,000 rpm for 1h at 4°C. The supernatant was collected and stored at -80°C until use. IL-1β, IL-1α, IL-2, IL-4, IL-5, IL-6, IL-7, IL-8, IL-10, IL-12p40/IL-23, IL-12p70, IL-15, IL-16, IL-17, Eotaxin, Eotaxin-2, Eotaxin-3, IP-10, MCP-1, MCP-2, MCP-3, MCP-4, MDC, MIP-1α, MIP-1β, TARC, GM-CSF, TNF-β, VEGF-A, and IFN-γ were quantified in fecal supernatants using human multiplex immunoassays [Meso Scale Discovery (MSD), Rockville, MD] according to the manufacturer’s protocol. Each fecal lysate was tested in duplicate. Plates were read using the QuickPlex SQ 120 (MSD, Rockville, MD). The concentration of each analyte was determined using standard calibrators and analyzed using the Meso Scale Discovery Workbench software v15.0 (MSD, Rockville, MD).

### Fecal DNA Extraction, Metagenomic Library Construction and Sequencing

Genomic DNA was extracted from homogenized fecal samples stored in DNA/RNA shields using the fecal/soil microbe kits (Zymo, Irvine, CA) according to the manufacturer’s instructions. Briefly, a 500 µl aliquot of fecal material mixture was homogenized using bead lysis and centrifuged down to remove lysate debris. Metagenomic sequencing libraries were constructed from the same DNA using Illumina Nextera XT flex kit according to the manufacturer recommendations. Libraries were then pooled together in equimolar proportions and sequenced on a single Illumina NovaSeq 6000 S2 flow cell at the Genomic Resource Center at the University of Maryland School of Medicine.

### Intestinal Microbiome Analyses

Metagenomic sequence data were pre-processed using the following steps: 1) human sequence reads and rRNA LSU/SSU reads were removed using BMTagger v3.101 (100) using a standard human genome reference (GRCh37.p5) (101); 2) rRNA sequence reads were removed *in silico* by aligning all reads using Bowtie v1 (102) to the SILVA PARC ribosomal-subunit sequence database (103) (sequence read pairs were removed even if only one of the reads matched to the human genome reference or to rRNA); 3) the Illumina adapter was trimmed using Trimmomatic (104); 4) sequence reads with average quality greater than Q15 over a sliding window of 4 bp were trimmed before the window, assessed for length, and removed if less than 75% of the original length; and 5) no ambiguous base pairs were allowed. The taxonomic composition of the microbiomes was established using MetaPhlAn version 2 (40). A heatmap was constructed from the 25 most abundant intestinal bacterial taxa measured by relative abundance in samples collected from 38 preterm infants enrolled in the study. Ward linkage clustering was used to cluster samples based on their Jensen-Shannon distance calculated in vegan package in R (42). The number of clusters was validated using gap statistics implemented in the *cluster* package in R (43) by calculating the goodness of clustering measure. Significance value for cytokine abundance in comparing categories was calculated using Wilcoxon rank sum test using *ggsignif* R package (41). Metagenomics dataset was mapped to the protein database UniRef90 (105) to ensure comprehensiveness in functional annotation, and was then summarized using HUMAnN2 (Human Microbiome Project Unified Metabolic Analysis Network) (v0.11.2) (106), which efficiently and accurately determines the presence, absence, and abundance of metabolic pathways in a microbial community. Further, HUMAnN2 employed a tiered search strategy that enables a species-resolved functional profiling of metagenomes to characterize the contribution to the functional pathways of a known species. Canonical Correspondence Analysis (CCA) was used in ordination analysis of the taxonomic profiles and cytokine profiles. Biplot was generated using vegan package (51, 107) based on the square root of bray-curtis distance. CA1 and CA2 are selected as the major components based on the eigenvalue. A species score was scaled proportional to the eigenvalues, representing the direction from the origin of a group having a larger than average value for the particular species (42, 51). The species scores greater than 1 are used to select the species that were considered the most significant contributors to each group. Only species/genera significantly (p value < 0.05) associated with at least one cytokine response were included. All species/genera were required to be detected in at least 1% of more than one sample. Plot was generated using R package ‘complexheatmap’ (47).

## Data Availability Statement

All metagenomic data were deposited in experiment ID SRX12885186 to SRX12885247 under BioProject PRJNA776332 (https://www.ncbi.nlm.nih.gov/bioproject/PRJNA776332) for open assessment. Other information, please refer to **Supplementary Material**.

## Ethics Statement

This study was approved and carried out in accordance with protocols approved by the institutional review boards of the University of Maryland School of Medicine and Mercy Medical Center. Written informed parental consent was obtained for the participation of all infants in the study, in accordance with approved protocol UMB HP-00049647.

## Author Contributions

JML-D, MFP, and BM designed the research. SS, HL, CH-C, and RV conducted the clinical study and sample preparation. YS and BM performed the microbiome and statistical analyses. JML-D performed the cytokine and chemokine measurements. JML-D, JR, MFP, and BM wrote the paper. All authors contributed to the article and approved the submitted version.

## Funding

This study was supported, in part, by The Gerber Foundation award (PID 6361), the National Institute of Diabetes and Digestive and Kidney Diseases (NIDDK) of the National Institutes of Health award number R21DK123674 (to BM), the National Institute of Allergy and Infectious Diseases (NIAID) of the National Institute of Health awards numbers U19AI145825 and P01AI125181 Immunology Core (to MFP), and the Institute for Clinical and Translational Research (ICTR) at University of Maryland Accelerated Translational Incubator Pilot (ATIP) award (PID 11).

## Conflict of Interest

The authors declare that the research was conducted in the absence of any commercial or financial relationships that could be construed as a potential conflict of interest.

## Publisher’s Note

All claims expressed in this article are solely those of the authors and do not necessarily represent those of their affiliated organizations, or those of the publisher, the editors and the reviewers. Any product that may be evaluated in this article, or claim that may be made by its manufacturer, is not guaranteed or endorsed by the publisher.
