# Supplementary material for: Altered Gut Microbiome and Fecal Immune Phenotype in Early Preterm Infants With Leaky Gut
Source: Front Immunol. 2022 Feb 23;13:815046. doi: 10.3389/fimmu.2022.815046 (PMC8905226; doi:10.3389/fimmu.2022.815046)
Supplement: Supplementary file 1 [file DataSheet_1.docx]

**Supplemental Figure 1.** Boxplot of the community diversity levels of different microbiota types. Plotted are interquartile ranges (IQRs, boxes), medians (line in box), and mean (red diamond). Significance value was calculated using Wilcoxon rank sum test using *ggsignif* R package (Ahlmann-Eltze, 2017). Asterisk (*) denotes the level of significance. Microbiota type was assigned according to the clustering pattern demonstrated in **Figure 1**.

**Supplemental Figure 2.** Boxplot of the cytokine levels of different microbiota types. Plotted are interquartile ranges (IQRs, boxes), medians (line in box), and mean (red diamond). Significance value was calculated using Wilcoxon rank sum test using *ggsignif* R package (Ahlmann-Eltze, 2017). Star sign (*) denotes the level of significance. Microbiota type was assigned according to the clustering pattern demonstrated in **Figure 1**.

**Supplemental Figure 3.** Summary of species and genus associations with cytokine responses using Spearman correlation (p value < 0.05). All species/genera were required to be detected in at least 1% of more than one sample. Only species/genera significantly (p value < 0.05) associated with at least one cytokine response are displayed using correlation coefficiency. Plot was generated using R package ‘complexheatmap’ (Gu et al., 2016).

**Supplemental Figure 4.** Canonical Correspondence Analysis (CCA) of the functional pathways of microbiome using: **A**) the unstratified profile from microbial taxonomic groups, to characterize the overall contribution of the species to the functional potential; **B**) stratified profiles of the functional pathways from contributing taxonomic groups, to characterize the specific pathways and the contributing species. CCA was based on bray-curtis distance. CA1 and CA2 selected as the major components based on the eigenvalue. The microbial functional pathways were characterized using HUMAnN2 (v0.11.2)(Franzosa et al., 2018) for both species-stratified and unstratified profiles of the functional pathways. Uniref90 database (Kanehisa et al., 2012) was used as the reference. A scaled eigenvalues was shown on the plot to represent the direction from the origin where a group has a larger than average value for the particular profile (Oksanen et al., 2011; Jari Oksanen, 2016). * Microbiota type was assigned according to the clustering pattern demonstrated in **Figure 1**.

**Supplemental Figure 5.** Boxplot of the cytokine levels of different categories of delivery mode. **ABBR**: SVD: spontaneous vaginal delivery; CS: cesarean section. Plotted are interquartile ranges (IQRs, boxes), medians (line in box), and mean (red diamond). Significance value was calculated using Wilcoxon rank sum test using *ggsignif* R package (Ahlmann-Eltze, 2017). Star sign (*) denotes the level of significance.

**Supplemental Table 1.** Clinical information of the 40 early preterm infant subjects used in this study.

**Supplemental Table 2.** Statistics of the metagenome sequencing of fecal samples from 38 subjects.

**Supplemental Table 3.** Taxonomic profiles of the 61 fecal specimens collected from 38 subjects during 7-10 postnatal days when intestinal permeability was measured. The taxonomic composition of the microbiomes was established using MetaPhlAn version 2 (Segata et al., 2012).

**Supplemental Table 4.** Statistics of Canonical Correspondence Analysis (CCA). CA1 and CA2 are selected as the major components based on the eigenvalue. CCA was generated using vegan package (Dixon, 2003; Jari Oksanen, 2016) based on the square root value of bray curtis distance of **A**) taxonomic profiles; **B**) cytokine and chemokine profiles; microbial functional pathway profiles of **C**) all species for which the functional pathways

were integrated/unstratified and **D**) individual functional ways identified/stratified by a contributing species given multiple species could contribute to the same pathway.

**Supplemental Table 5.** Cytokine and chemokine measurements of fecal lysates. Markers were quantified using human multiplex immunoassays.

**Supplemental Figure 1.**


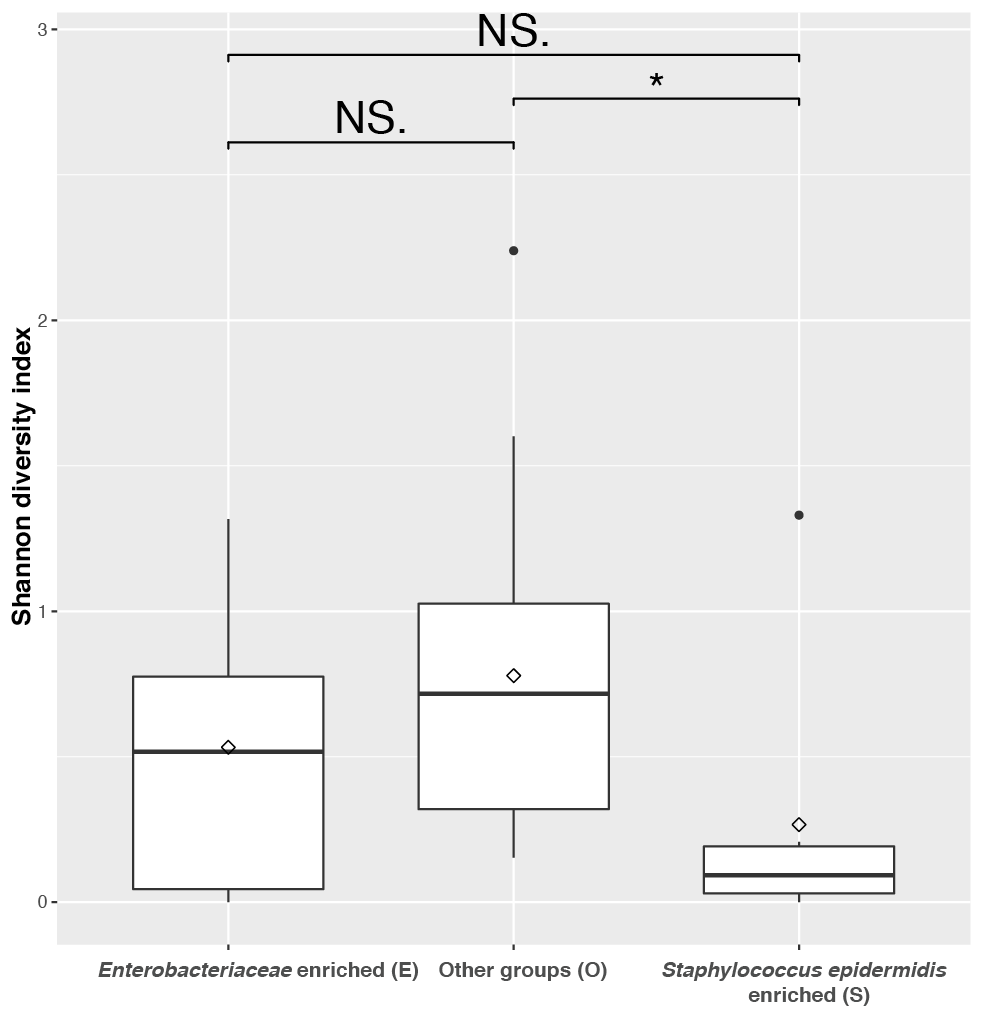


**Supplemental Figure 2.**


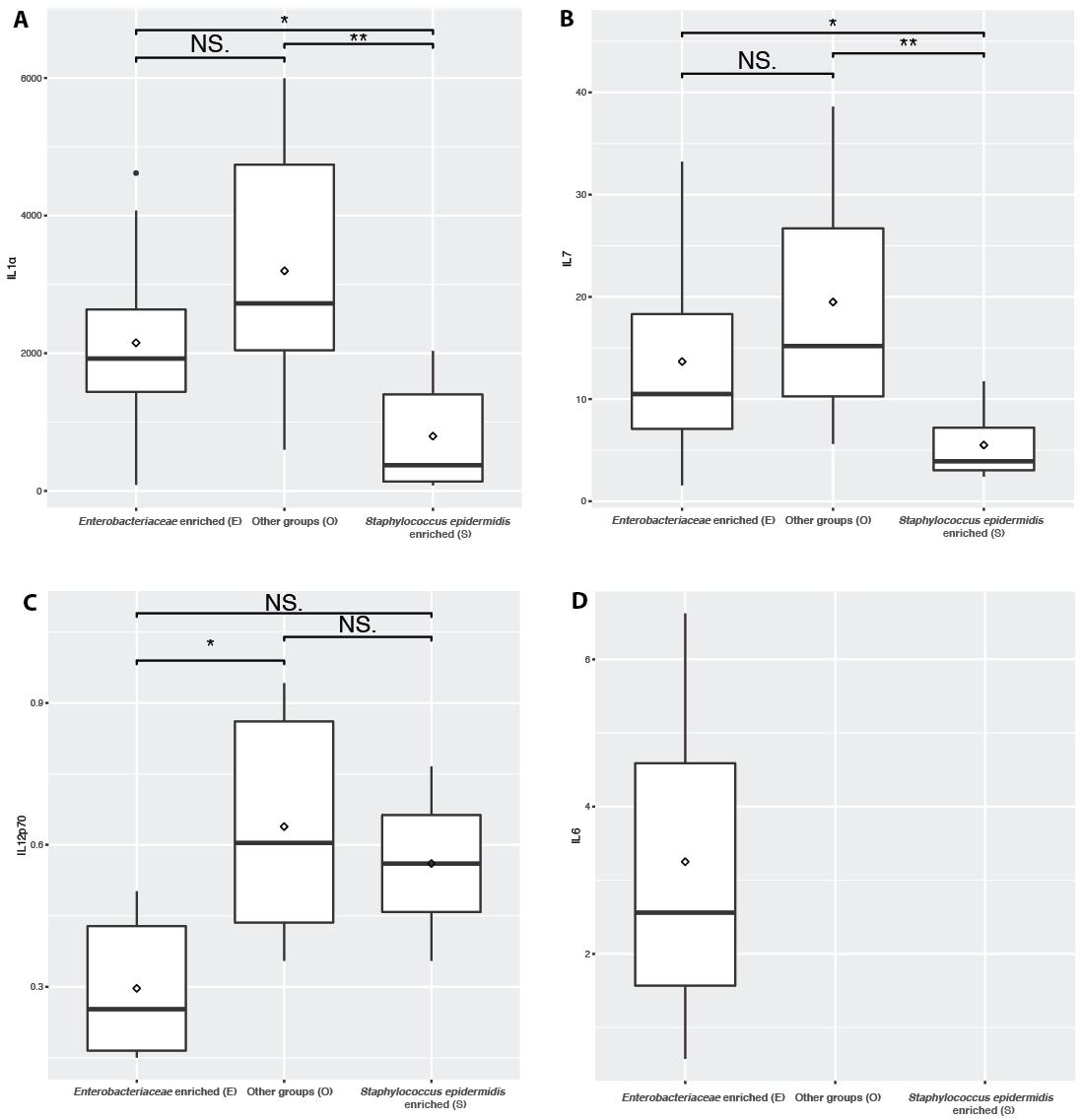


**Supplemental Figure 3.**

**
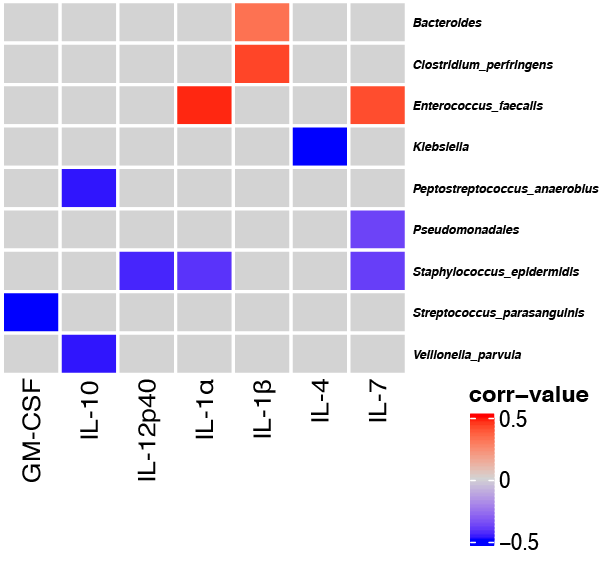
**

**Supplemental Figure 4**

**
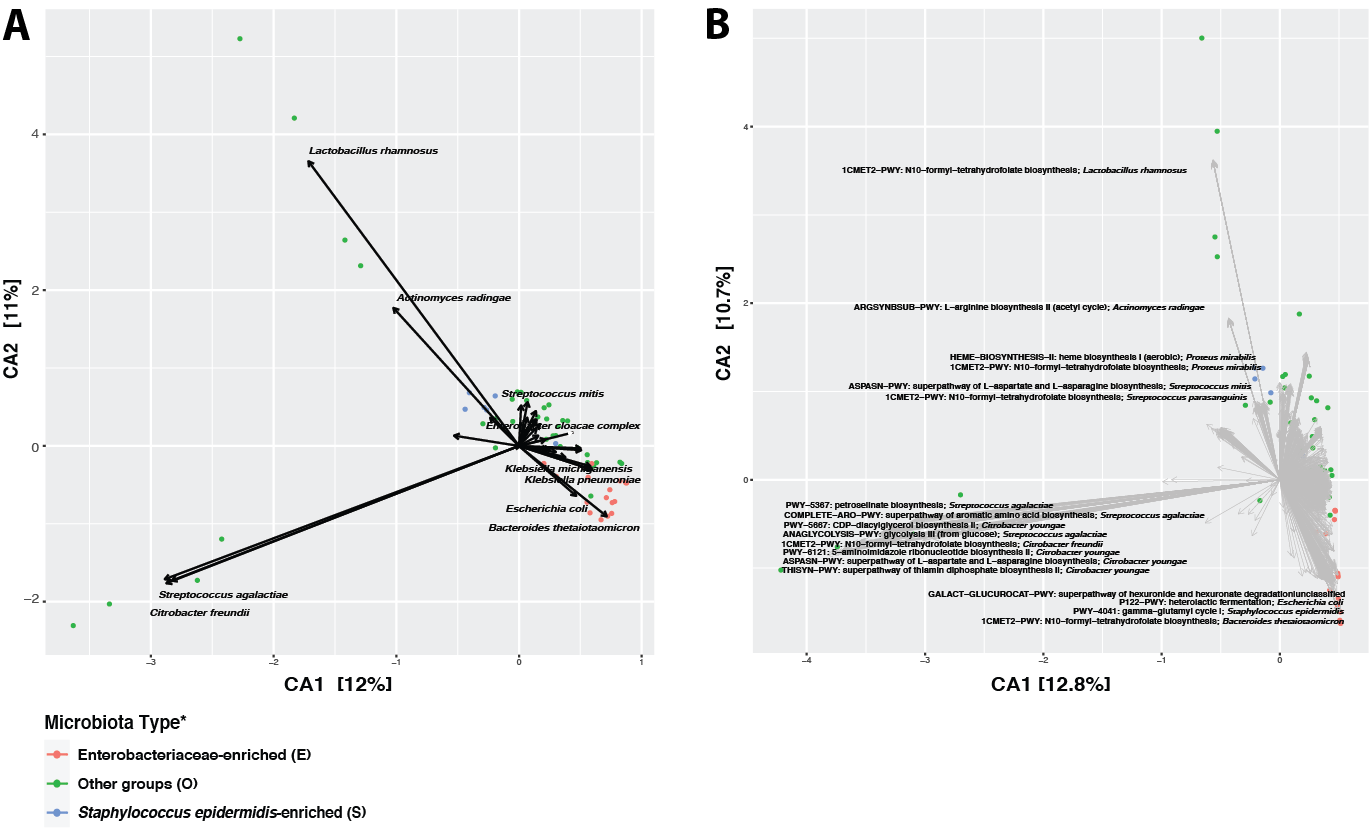
**

**Supplemental Figure 5.**

**
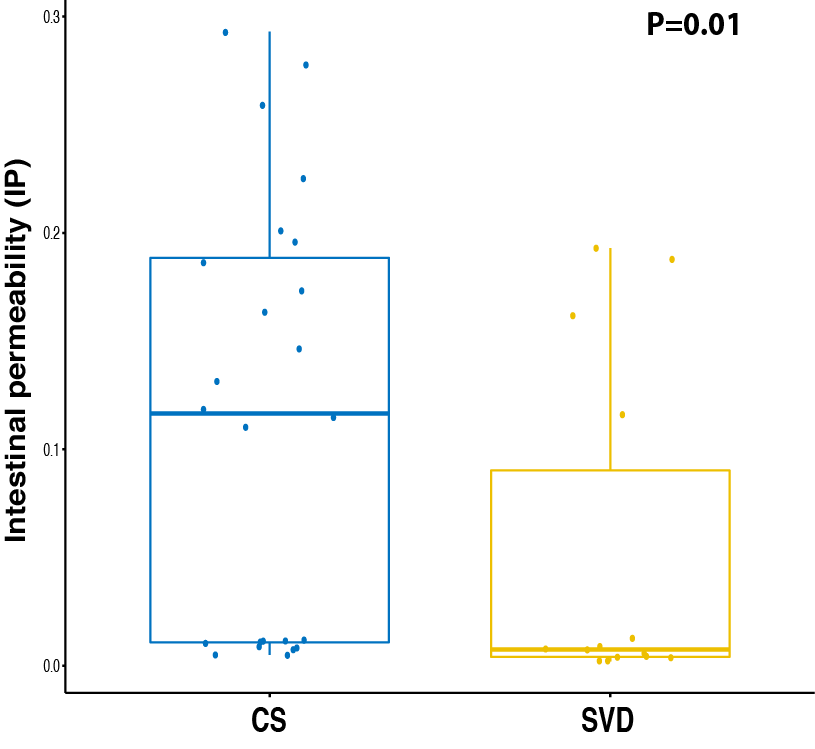
**

# References:

Ahlmann-Eltze, C. (2017). *ggsignif* [Online]. Available: <https://github.com/const-ae/ggsignif> [Accessed June 16, 2018].

Dixon, P. (2003). VEGAN, a package of R functions for community ecology. *Journal of Vegetation Science* 14(6)**,** 927-930.

Franzosa, E.A., McIver, L.J., Rahnavard, G., Thompson, L.R., Schirmer, M., Weingart, G., et al. (2018). Species-level functional profiling of metagenomes and metatranscriptomes. *Nat Methods* 15(11)**,** 962-968. doi: 10.1038/s41592-018-0176-y.

Gu, Z., Eils, R., and Schlesner, M. (2016). Complex heatmaps reveal patterns and correlations in multidimensional genomic data. *Bioinformatics* 32(18)**,** 2847-2849. doi: 10.1093/bioinformatics/btw313.

Jari Oksanen, F.G.B., Michael Friendly, Roeland Kindt, Pierre Legendre, Dan McGlinn, Peter R. Minchin, R. B. O'Hara, Gavin L. Simpson, Peter Solymos, M. Henry H. Stevens, Eduard Szoecs and Helene Wagner (2016). "vegan: Community Ecology Package. R package". version 2.4-1. ed.).

Kanehisa, M., Goto, S., Sato, Y., Furumichi, M., and Tanabe, M. (2012). KEGG for integration and interpretation of large-scale molecular data sets. *Nucleic acids research* 40(Database issue)**,** D109-114. doi: 10.1093/nar/gkr988.

Oksanen, J., Blanchet, F.G., Kindt, R., Legendre, P., Minchin, P.R., O'Hara, R.B., et al. (2011). vegan: Community Ecology Package. *R package version 2.0-2.*

Segata, N., Waldron, L., Ballarini, A., Narasimhan, V., Jousson, O., and Huttenhower, C. (2012). Metagenomic microbial community profiling using unique clade-specific marker genes. *Nature methods* 9(8)**,** 811-814. doi: 10.1038/nmeth.2066.
